# KLOTHO polymorphisms and age-related outcomes in community-dwelling older subjects: The São Paulo Ageing & Health (SPAH) Study

**DOI:** 10.1038/s41598-020-65441-y

**Published:** 2020-05-22

**Authors:** Rosa Maria R. Pereira, Thiago Quadrante Freitas, André Silva Franco, Liliam Takayama, Valeria F. Caparbo, Diogo S. Domiciano, Luana G. Machado, Camille P. Figueiredo, Paulo R. Menezes, Luiz Fernando Onuchic, Isac de Castro

**Affiliations:** 1 Bone Metabolism Laboratory, Rheumatology Division, Faculdade de Medicina FMUSP, Universidade de Sao Paulo, Sao Paulo, SP Brazil; 2 Department of Preventive Medicine, Faculdade de Medicina FMUSP, Universidade de Sao Paulo, Sao Paulo, SP Brazil; 3 Divisions of Molecular Medicine and Nephrology, Faculdade de Medicina FMUSP, Universidade de Sao Paulo, Sao Paulo, SP Brazil

**Keywords:** Genetics research, Predictive markers

## Abstract

Defective *KLOTHO* gene expression in mice led to a syndrome resembling human ageing. This study evaluated three *KLOTHO* polymorphisms, namely G395A, C1818T, and C370S, in an elderly population (mean age of 73 years) and their associations with ageing-related outcomes (cardiovascular events, kidney function, osteoporosis, sarcopenia) and mortality. Estimated glomerular filtration rates (eGFR) was lower in subjects with 1818TT (P = 0.047) and 370SS (P = 0.046) genotypes. The 1818TT genotype (P = 0.006) and 1818T allele were associated with higher frequency of myocardial infarction (MI) (CC:1.7% vs. CT + TT:7.0%; P = 0.002). The 370SS genotype was associated with lower stroke frequency (P = 0.001). MI (OR 3.35 [95% CI: 1.29–8.74]) and stroke (OR 3.64 [95% CI: 1.48–8.97]) were associated with mortality. Regarding MI, logistic regression showed 1818T allele was a risk factor for death-related MI (OR 4.29 [95% CI: 1.60–11.52]; P = 0.003), while 370C was protective (OR 0.03 [95% CI: 0.01–0.08]; P < 0.001). Regarding stroke, the 395A and 370C alleles were protective factors (respectively: OR 0.28 [95% CI: 0.20–0.80]; P = 0.018; OR 0.10 [95% CI: 0.05–0.18]; P < 0.001). This is the first study to determine potential associations between common ageing-related outcomes/mortality and *KLOTHO* polymorphisms. The 1818T allele was a risk factor for MI-related death. The 395A and 370C alleles were protective factors for stroke-related death in elderly from community.

## Introduction

The percentage of elderly people is increasing more than any other age group worldwide^1^. This trend comes with significant social consequences: communities with higher percentages of elderly people present higher disease rates (especially of chronic, noncommunicable diseases) and increased dependency of residential and hospital care^1^. Under these circumstances, several studies directed to basic health fields decided to look for a deeper comprehension of human aging to subsequently focus on intervention strategies^2,3^.

The *KLOTHO* gene was discovered in 1997, when a study revealed that its defective expression in mice led to a syndrome that resembles human ageing, including a short lifespan, arteriosclerosis, and osteoporosis^4^. Its products exist as a secreted protein^5^ or a transmembrane protein expressed predominantly in the distal renal tubules, choroid plexus, and pituitary gland^4^. Their function in humans, however, remains unknown. The secreted form suppresses oxidative stress and growth factor signalling, including insulin/IGF-1 signalling, all effects associated with longer life span^6^.

At least 10 *KLOTHO* mutations and single-nucleotide polymorphisms (SNPs) have been described in the humans. Nonetheless, the effects of each SNP in human ageing are still unclear. In fact, studies in human populations have sought for associations between aging, longevity, and some of such variants, including the most common SNPs G395A and C1818T^7^ and the functional Klotho variant KL-VS. This last variant comprises six polymorphisms in linkage disequilibrium, two of which resulting in amino acid substitutions: F352V and C370S^6^. Associations between these variants and age-related outcomes were demonstrated in different populations, mainly in Asians^8,9^.

There is only one publication regarding KLOTHO polymorphisms and age-related comorbidities in a Brazilian population^10^. Hence, the present study seeks to evaluate three KLOTHO gene polymorphisms in an elderly population from the São Paulo Ageing & Health Study (SPAH) and their potential association mainly with mortality, but also with the prevalence of major ageing-related outcomes, including osteoporosis, osteoporotic fractures (clinical and vertebral fractures), sarcopenia, kidney function (estimated glomerular filtration rate [eGFR]), and cardiovascular events (i.e. myocardial infarction and stroke).

## Results

The demographic, anthropometric and clinical data of the 601 subjects are shown in Table 1, including genotype frequencies. The mean population age was 73.1 years. Body mass index varied from 17.2 to 47.4, with a mean of 28.1 kg/m².

**Table 1:** Baseline demographic, genetic, anthropometric, risk factors for osteoporosis, cardiovascular events, and image data from 601 elderly subjects from the São Paulo Ageing & Health Study (SPAH).

		Mean or Absolute frequency N = 601	SD or %
Age (years)		73.1	5.4
Sex (n)	Female	381	63.4%
Ethnicity (n)	Caucasian	379	63.1%
Weight (kg)		67.3	12.8
BMI (kg/m²)		28.1	4.8
Smoking history (n)		70	11.6%
Alcohol intake (n)		91	15.1%
Low physical activity (n)		557	92.7%
Aortic calcification (n)		346	57.6%
Calcification score (points)		5	6.01
eGFR (mL/min)		59.2	18.5
Osteoporosis (n)		281	46.8%
Osteopenia (n)		247	41.1%
Falls (n)		202	33.6%
Spontaneous fracture (n)		66	11%
Vertebral fracture (n)		173	28.8%
Low Appendicular Muscle Mass (n)		96	16%
Cardiovascular events (n)	**Total**	80	13.3%
	**Angina**	25	4.2%
	**MI**	27	4.5%
	**Stroke**	30	5%
C1818T (n)	**CC**	288	47.9%
	**CT**	253	42.1%
	**TT**	60	0.1%
G395A (n)	**GG**	407	67.7%
	**GA**	183	30.5%
	**AA**	11	0.02%
C370S (n)	**CC**	467	77.7%
	**CS**	131	21.8%
	**SS**	3	0.005%

The data are expressed as mean or absolute frequency (n), and standard deviation (SD) or percentage (%).

### Cardiovascular events

Of the 601 subjects, 80 (13.3%) experienced cardiovascular events, including *angina pectoris*, myocardial infarction or stroke.

Only 27 (4.5%) subjects had a history of MI. The analysis of the C1818T SNP revealed that the 1818TT genotype was associated with a higher frequency of MI (P = 0.006) (Table 2). Single-allele frequency analysis also showed association between 1818T and higher MI frequency (CC 1.7% vs. CT + TT 7.0%; P = 0.002). G395A and C370S had no statistically significant association with MI.

**Table 2:** Frequencies of isolated genotypes of *KLOTHO* SNPs C1818T, G395A, and C370S in the patient groups with Myocardial Infarction, Stroke and Vascular Calcification at baseline.

C1818T (rs564481)	Myocardial Infarction		Stroke		Vascular calcification	
	n (%)	p value	n (%)	p value	n (%)	p value
CC	5 (1.7)	**0.006**	18 (6.2)	0.791	163 (64.2)	0.661
CT	17 (6.7)		11 (4.3)		144 (63.2)	
TT	5 (8.3)		1 (1.7)		39 (69.6)	
Allele C	22 (4.1)	0.130	29 (3.4)	0.649	307 (63.7)	0.379
Allele T	22 (7.0)	**0.002**	12 (3.8)	0.543	183 (64.4)	0.949
**G395A (rs1207568)**						
GG	18 (4.4)	0.758	26 (6.4)	0.455	242 (66.5)	0.285
GA	8 (4.4)		4 (2.2)		98 (59.4)	
AA	1 (9.1)		0 (0)		6 (66.7)	
Allele G	26 (4.4)	0.457	30 (5.1)	0.899	340 (64.4)	0.882
Allele A	9 (4.6)	0.905	4 (2.1)	0.133	104 (59.8)	0.128
**C370S (rs9527025)**						
CC	21 (4.5)	0.992	20 (4.3)	**<0.001**	270 (65.5)	0.468
CS	6 (4.0)		9 (6.8)		74 (59.7)	
SS	0 (0)		0 (0)		1 (100.0)	
Allele C	27 (4.5)	0.706	29 (4.9)	0.984	344 (64.3)	0.932
Allele S	6 (4.5)	0.992	9 (7.5)	0.246	75 (60.3)	0.285

Data expressed as absolute frequency (n) and percentage (%)

Analysed by Pearson χ^2^.

Thirty (5%) stroke events occurred in the studied population. The 370SS genotype was associated with a lower outcome frequency (P = 0.001) while single-allele analysis detected no significant differences (Table 2).

### Aortic Calcification

Three hundred and forty-six (57.6%) subjects presented with abdominal aortic calcification score >0 visible on radiographic assessment. However, none of the three polymorphisms were associated with this outcome (Table 2).

### Estimated glomerular filtration rate (eGFR)

Subjects with the 1818TT and 370SS genotypes featured lower eGFR (P = 0.047 and P = 0.046, respectively) (Table 3). Furthermore, the presence of 1818C allele was associated with higher eGFR (TT: 55.5 vs. CC + CT: 58.1; P = 0.033). Single-allele frequency analysis directed to the C370S and G395A SNPs, however, detected no significant associations.

**Table 3:** Isolated genotypes of *KLOTHO* SNPs C1818T, G395A, and C370S and respective values of estimated glomerular filtration rate (eGFR).

C1818T (rs564481)	eGFR	
	mL/min/1.73 m²	P value
CC	58.0 (45.8–70.5)	**0.047**
CT	58.2 (47.0–73.0)	
TT	55.5 (41.5–64.2)	
Allele C	58.1 (46.3–71.2)	**0.033**
Allele T	57.8 (45.8–70.8)	0.892
**G395A (rs1207568)**		
GG	57.7 (45.9–70.5)	0.468
GA	58.2 (44.7–71.6)	
AA	52.7 (40.9–69.9)	
Allele G	58.0 (45.9–70.6)	0.461
Allele A	58.1 (44.5–71.3)	0.814
**C370S (rs9527025)**		
CC	57.3 (45.2–69.3)	**0.046**
CS	60.1 (47.4–74.4)	
SS	50.0 (50.0–50.0)	
Allele C	57.9 (45.8–70.6)	0.698
Allele S	60.1 (47.7–74.5)	**0.039**

Continuous data are expressed as Median and Quartiles and analysed by Kruskal-Wallis Test adjusted by Bonferroni. For two groups, the analysis was performed by Mann-Whitney Test.

### Mortality

From 2005 to 2012, there were 51 (8.5%) deaths among the 601 participants, mostly related to cardiovascular events (15 [29.4%, 9 from stroke and 6 from MI]), followed by pulmonary non-neoplastic causes (COPD, pneumonia, fibrosis) (10, 19.6%), non-ischemic heart failure (8, 15.7%), infections (excluding pneumonia) (7, 13.7%), cancer (4, 7.8%), and other causes, including pulmonary thromboembolism, Alzheimer’s Disease and kidney failure (7, 13.7%). The mean age of the deceased was 79.7 years, with a standard deviation of 6.8 years.

MI and stroke were the only variables associated with mortality. They were therefore analysed by logistic regression, which was adjusted by sex, BMI and age considering the average life expectancy of the Brazilian population during the data collection period. No association was observed concerning other outcomes, including eGFR.

MI was independently associated with death, with an Odds Ratio (OR) of 3.35 [95%CI: 1.29–8.74]). The presence of 1818T was a risk factor for MI-related death OR: 4.29 [95%CI: 1.60–11.52]; P = 0.003), whereas both the presence of 370C and the female sex were independent protective factors (respectively: OR: 0.4 [95%CI: 0.2–1.0], P = 0.041; and OR: 0.03 [95% CI: 0.01–0.08]; P < 0.001) (Table 4).

**Table 4:** Logistic regression for death related to myocardial infarction and stroke.

	P value	Odds Ratio adjusted	Inferior 95% C.I.	Superior 95% C.I.
**Logistic regression for Myocardial Infarction**				
Allele 1818T (presence)	0.004	4.3	1.6	11.5
Allele 370C (presence)	<0.001	0.03	0.01	0.08
Sex (adjusted for male sex)	0.041	0.4	0.2	1.0
Age ≥ 73.5 years old	0.28	0.62	0.27	1.46
BMI ≥ 30 kg/m²	0.32	1.5	0.7	3.6
**Logistic regression for Stroke**				
Allele 395A	0.012	0.3	0.1	0.7
Allele 370C	<0.001	0.1	0	0.1
Sedentary behaviour	<0.001	7.0	2.9	17
Sex (adjusted)	0.749	0.9	0.4	1.9
BMI ≥ 30 kg/m²	0.14	1.8	0.8	4.1

Stroke was also related to death (OR 3.64 [95%CI: 1.48–8.97]), and multiple logistic regression found that 395A and 370C alleles were protective factors (respectively: OR 0.28 [95%CI: 0.20–0.80]; P = 0.018, and OR 0.10 [95%CI: 0.05–0.18]; P < 0.001) (Table 4).

### Other outcomes

No statistically significant associations between C1818T, G395A or C370S and lifestyle, including smoking history, alcohol intake and physical activity were found. Moreover, no polymorphism was independently associated with osteopenia/osteoporosis, clinical and vertebral fractures, and low appendicular muscle mass. (Supplementary Tables S1 and S2).

## Discussion

To the best of our knowledge, this is the first study to investigate potential associations between the most common ageing-related outcomes and mortality with *KLOTHO* polymorphisms, namely C1818T, G395A, and C370S, in community-dwelling subjects.

In this community population, cardiovascular events were the main cause of death, and, as expected, the female sex was a protective factor for MI-related death. The 1818T allele was associated with a higher myocardial infarction frequency and, according to the logistic regression analysis, constitutes a risk factor for MI-related death. Most studies analysed this polymorphism regarding cardiovascular disease using coronary angiography^8,11–14^, myocardial scintigraphy^15^ or coronary artery calcification^16^, mainly in Asian population^8,13,14,17,18^. Rhee et al. showed that individuals that harbour the 1818T allele are associated with a lower prevalence of coronary artery disease (CAD) than those with the CC genotype, particularly in younger Korean subjects (<60 years)^8^. Analysis of an Iranian population, in turn, revealed a protective effect of the 1818CC genotype for age-related occurrence of CAD combined with hypertension in the subgroup older than 57 years^12^. The only study conducted in a Brazilian population so far, however, did not analyse the C1818T polymorphism in the mentioned setting^10^.

The other two KLOTHO polymorphisms analysed (G395A and C370S) were not associated with MI. Regarding G395A SNP, previous studies showed an association with CAD mainly in Asian population^13,14,17,19^, and a Spanish study^20^ found that it was a predictor of diabetes. On the other hand, regarding C370S, studies from Massachusetts, USA revealed no association between C370S and cardiovascular disease (premature acute coronary artery disease and vascular calcification) in young^11^ and older patients^16^. Moreover, there are other evidences tying Klotho to cardiovascular events, even though not related to its polymorphisms, but to its serum and tissue expressions. In fact, a Brazilian community-dwelling elderly population showed association between serum Klotho levels and history of myocardial infarction^10^. A Spanish study found association between the vascular levels of Klotho expression and presence of cardiovascular disease and cardiovascular risk factors. Indeed, individuals with coronary artery disease had a significantly lower expression of Klotho in aortic samples^20^.

In the present study, stroke was an important cause of cardiovascular death. The multivariate analysis indicated that the S allele of the C370S SNP and the A allele of the G395A SNP were protective factors for stroke-related death, whereas sedentary behaviour was identified as a risk factor for this outcome. Prior data have demonstrated that homozygous 370CC or 370SS individuals have a higher risk for stroke, while heterozygotes display a lower risk^21,22^. The C1818T SNP was also studied in Indian population, but was not associated with ischemic stroke^21^. Furthermore, in Korean women, the G395A SNP A allele was related to cardioembolic stroke^23^.

The present study found no associations between vascular calcification and Klotho polymorphisms in our community-dwelling elderly subjects. Of note, this issue had never been investigated in humans.

Renal dysfunction has been related to Klotho protein, but there are few studies correlating kidney function to *KLOTHO* polymorphisms in subjects without chronic kidney disease. Our results showed that 1818TT and 370CC genotypes were associated with lower eGFR. Analysis of two Chinese populations (Uygur and Kazak) with different longevities revealed that such genotypes were associated with higher serum creatinine in the Kazak population, which has shorter longevity^5^.

No association between Klotho polymorphisms and BMD and fractures were found in the present study. In contrast, data from literature have speculated that Klotho may have an effect on osteocytes^24,25^ and osteoblasts^26^, in addition to promoting osteoclast differentiation^24^. Data from previous studies are controversial, showing that the CS/SS genotypes (C370S SNP) were associated with higher BMD, but not with osteoporotic fractures in Spanish women^27^ and young/middle-aged Spanish men^28^. Moreover, postmenopausal white and Japanese women with the G395A SNP GG genotype tended to have lower BMD^29^, while A allele carriers among Japanese women had higher BMD^9,30^. The same association was found with T allele carriers of the C1818T SNP in postmenopausal women^30^, a finding not observed in another report^9^.

Potential associations between sarcopenia and *KLOTHO* polymorphisms had not been previously studied before, so the lack of such associations revealed by our study consists in negative but novel information.

Klotho polymorphisms have been previously associated with longevity (defined, depending on the study, as people over the age of 66, 75 or 93 years) in different populations^6,31,32^. Such findings prompted research about those variants and mortality itself, however only in chronically ill populations, such as patients in haemodialysis^33,34^ and IgA nephropathy^35^. Our study is the first one to examine *KLOTHO* SNPs and mortality in elderly community-dwelling subjects and two polymorphisms were associated with the main events that led to death, i.e., stroke and myocardial infarction.

## Materials and Methods

### Subjects

This study was based on data from the São Paulo Ageing & Health (SPAH) Study, which surveyed 65-year-old and older individuals living in a community in the Butantã district, located on the western zone of the city of São Paulo. The first survey took place from 2005 to 2007, and provided the baseline data^36^. The subjects were invited to participate in a second survey from 2010 to 2012, which provided data regarding mortality^37^. Full details pertaining to the study population, assessments, and procedures have been previously reported elsewhere^36,37^.

One hundred and twenty-five participants were interviewed on the first survey; 725 (70.73%) attended the second evaluation, and 601 of them (381 women and 220 men) had blood samples collected for DNA extraction and were included in the genotype-based analyses. Fifty-one of the analysed subjects (8.49%) died from 2005 to 2012.

This study was approved by the local ethics committee (Comissão de Ética para Análise de Projetos de Pesquisa do HCFMUSP [CAPPesq] #426/06), and all the participants provided written informed consent. All the methods were carried out in accordance with relevant guidelines and regulations stated by Declaration of Helsinki.

### Data collection and assessments

Each subject was interviewed by a physician and responded to a standard questionnaire designed to gather information about lifestyle, health behaviours, and medical history, including age, smoking status, physical activity (ability to perform daily chores), alcohol consumption (at least 3 units per day), previous clinical fragility fractures, history of falls during the past year, and history of myocardial infarction or stroke.

Baseline measurements included height and weight, and were carried out using standard protocols to calculate the body mass index (BMI) and the appendicular skeletal muscle mass (ASM). Baseline laboratory and imaging evaluations included thoracic and lumbar spine radiographs, bone mineral density (BMD) measurement^36,38^, and serum creatinine concentration, determined using standard automated laboratory methods. Estimated glomerular filtration rate (eGFR) was calculated according to the Modification of Diet in Renal Disease (MDRD) formula^39,40^. The second interview provided data concerning mortality.

### BMD measurement and Osteoporosis classification

Bone Mineral Density was measured using dual-energy X-ray absorptiometry (DXA, Hologic Inc. Bedford, MA, USA) at the lumbar spine, femoral neck, and total hip. All BMD measurements were performed by the same experienced technologist, following the recommendations by the International Society for Clinical Densitometry (ISCD)^41^. The least significant change was calculated with 95% confidence to be 0.033 g/cm^2^ for anteroposterior spine, 0.047 g/cm^2^ for femoral neck, and 0.039 g/cm^2^ for total hip. BMD characterization was performed according to the WHO classification: normal, T-score ≥ −1; osteopenia, T-score < −1 and > −2.5; and osteoporosis, T-score ≤ −2.5 in the spine, femoral neck, or total hip^41^.

### Clinical osteoporotic fractures and assessment of vertebral fracture

Osteoporotic fracture was defined as a fracture occurring at sites characteristic of bone fragility (e.g., the rib, spine, forearm, humerus, and femur) on fifty-year-olds and older. Fractures occurring in the face, skull, ankles, elbows, and fingers were not considered osteoporotic fractures^42^.

Standard lateral thoracic and lumbar spine radiographs were taken using a 40-in tube-to-film distance centered at T7 and L2. All images provided good visibility of all vertebrae from T4 to L4, and vertebrae could be reliably identified. Two experienced readers independently performed the identification of vertebral fractures. Each of the T4–L4 vertebral images was evaluated to detect fractures, and nonvisible vertebrae were excluded. The agreement rate between the readers, which was established on a random subsample of 60 radiographs, was 96% with a kappa coefficient of 0.82. A consensus was reached between the readers for any difference of interpretation. Vertebral fractures were classified using the Genant semiquantitative approach^43^.

### Appendicular skeletal muscle mass (ASM)

Appendicular skeletal muscle mass (ASM) was measured by DXA: ASM relative to height and total fat mass were assessed. Relative ASM was derived by adjusting for fat mass in addition to height^44^. Linear regression was used to determine the relationship between ASM relative to height (in meters) and fat mass (in kilograms). The residuals of the regression were used to identify those with ASM lower than the predicted value (given by an equation derived from the model), for an individual fat mass. A positive residual would indicate a relatively muscular individual, whereas negative values would indicate relatively sarcopenic individuals. In men, the equation resultant from the model was as follows: ASM (kg)  =   −20.67  +  22.48 × height (m)  +  0.177 × fat mass (kg); and, in women: ASM (kg) = −14.51 + 17.27 × height (m) + 0.20 × fat mass (kg). The 20th percentile of the distribution of residuals was used as the cut point for sarcopenia according to ASM adjusted for fat. The cut-off corresponded to a residual of −2.06 in men^45^, and −1.45 in women^46^ in our Brazilian sample.

### Assessment of abdominal aortic calcification

Lateral lumbar spine radiographs were acquired to quantify aortic calcification, besides assessing vertebral fractures. Calcium deposits were considered to be present if the densities were visible in an area that was parallel to the lumbar spine and anterior to the lower part of the spine. These calcific densities were graded on a 0 to 3 scale at each lumbar vertebrae segment: 0 denotes no aortic calcific deposits; 1 denotes small scattered calcific deposits that occupies less than one third of the longitudinal wall of the aorta; 2 indicates that one third or more but less than two thirds of the longitudinal wall of the aorta is calcified; and 3 indicates that two thirds or more of the longitudinal wall of the aorta is calcified^47^. This resulted in an abdominal aortic calcium score (AACS) ranging from 0 to 24. The reproducibility of AACS blind assessment was analysed using the Wilcoxon test for sum score as continuous and categorized data. In both cases, the *P* values were higher than 0.10 (*P*  =  0.129 for the continuous data analysis and *P*  =  0.99 for the categorized data analysis), confirming appropriate level of reproducibility.

### Cardiovascular event assessment

During the interview, when a past history of myocardial infarction (MI) or stroke was identified, the participant’s medical records (completed by the family doctor or hospital) were reviewed. Stroke was defined as a sudden focal neurological deficit of presumed vascular origin that persisted for more than 24 hours. MI was defined as ischemic symptoms, development of diagnostic Q waves on the electrocardiogram, electrocardiographic changes indicative of ischaemia (ST segment elevation or depression), or coronary artery intervention.

### Mortality data

Mortality data of the original SPAH cohort was gathered from the date of the baseline examination through December 31, 2012. They were collected from the publicly available death certificates ascertained by the Programa de Aprimoramento das Informações de Mortalidade no Município de São Paulo (PRO-AIM, Improvement Program of Information on Mortality in São Paulo). The underlying causes of death were coded according to the 10th revision of the International Classification of Diseases (ICD-10). Cardiovascular mortality was defined as ICD-10 codes I00 to I99 and non-cardiovascular mortality as all other causes of death.

### Genotyping

Genomic DNA was isolated from peripheral blood leukocytes using the salting-out methodology^48^, and was stored at −70 °C prior to analysis. The TaqMan allelic discrimination method was employed for genotyping, using specific probes labelled with VIC and FAM (Applied Biosystems, Foster City, CA, USA).

The analysed SNPs were chosen based on previous literature data that suggested possible associations with ageing and functional significance^5,7–9,49,50^. Such SNPs comprised C1818T (rs564481), a silent mutation^30^ located in exon 4 of *KLOTHO*^7,30^; G395A (rs1207568), located in the promoter region of *KLOTHO*^7,30^ and possibly associated with its expression and function^30^; and C370S (also named G1117C; rs9527025), located in exon 2^51^. C370S is one of the SNPs that compose the functional Klotho variant KL-VS, which impairs the protein function by influencing its trafficking and catalytic activity^51,52^.

All SNPs were genotyped using commercial TaqMan assays (assay IDs: rs1207568: C_7604792_10; rs9527025: C_2983036_20; rs564481: C_592739_10) with TaqMan Genotyping Master Mix and were measured using a StepOne plus detection system (Applied Biosystems). Cycling conditions included a denaturation step at 95 °C for 10 minutes followed by 40 cycles of denaturation at 92 °C for 15 seconds and annealing for 1 minute at 60 °C. To assess the genotyping quality, the polymorphisms were analysed twice in 5% of all samples, yielding a 98% concordance.

### Rationale for outcome selection

Following the evidence-based choice for studying each of these three *KLOTHO* SNPs, we selected the most clinically relevant ageing-related outcomes associated to them. As discussed above, there lacks data correlating mortality to *KLOTHO* SNPs, thus mortality is a hard outcome that was chosen to be one of this studies cornerstones. Potential causes for death, as cardiovascular events, and outcomes related to healthy ageing that were previously studied in relation to these and other *KLOTHO* SNPs (CKD, BMD, etc) were chosen according to previous literature, also debated on Discussion.

### Statistical analysis

Categorical data were expressed as absolute frequency (n) and relative frequency and analysed by Pearson χ^2^. Continuous variables were expressed as mean ± standard deviation for parametric data or median and quartiles for non-parametric data. When comparing three or more groups, nonparametric data were analysed using the Kruskal-Wallis test adjusted by the Bonferroni post-test, while the Mann-Whitney test was applied for comparisons between two groups. In the multivariate analysis, Logistic Regression, results were expressed as odds ratio and 95% confidence interval. Hosmer–Lemeshow goodness-of-fit test was applied to evaluate the calibration of logistic regression model and to check the importance of the discrepancy between observed and expected for Myocardial Infarction and Stroke. Forward stepwise selection procedure was used to select the variables significantly related to Myocardial Infarction and Stroke, as assessed by the Likelihood Ratio test. Odds ratios with 95% confidence intervals were calculated for statistically significant variables. A P-value <0.05 was considered statistically significant. The genotype distribution and allele frequencies of the C1818T and G395A SNPs were consistent with the Hardy-Weinberg equilibrium (χ²= 0.16 with P = 0.68, and χ² = 3.49 with P = 0.06, respectively), whereas the ones associated with C370S were not (χ² = 3.90 with P = 0.05). However, since the number of subjects that would have to be excluded from the analysis to adjust it was higher than 5 to 10%, all the data were analysed to avoid additional bias^53^. Furthermore, for the allelic analyses, the number of patients was not duplicated as not to create bias from the duplication of phenotypic traits.

## Supplementary information

Supplementary information.
