# Supplementary material for: KLOTHO polymorphisms and age-related outcomes in community-dwelling older subjects: The São Paulo Ageing & Health (SPAH) Study
Source: Sci Rep. 2020 May 22;10:8574. doi: 10.1038/s41598-020-65441-y (PMC7244540; doi:10.1038/s41598-020-65441-y)
Supplement: Supplementary file 1 — Supplementary information. [file 41598_2020_65441_MOESM1_ESM.docx]

**KLOTHO polymorphisms and age-related outcomes in community-dwelling older subjects: São Paulo Ageing & Health Study (SPAH)**

**Rosa Maria R. Pereira^1,*^, Thiago Quadrante Freitas^1^, André Silva Franco^1^, Liliam Takayama^1^, Valeria F Caparbo^1^, Diogo S Domiciano^1^, Luana G Machado^1^, Camille P Figueiredo^1^, Paulo R Menezes^2^, Luiz Fernando Onuchic^3^, Isac de Castro^3^**

1. Bone Metabolism Laboratory, Rheumatology Division, Faculdade de Medicina FMUSP, Universidade de Sao Paulo, Sao Paulo, SP, Brazil.
2. Department of Preventive Medicine, Faculdade de Medicina da Universidade de Sao Paulo, Sao Paulo, SP, Brazil.
3. Divisions of Molecular Medicine and Nephrology, Faculdade de Medicina FMUSP, Universidade de Sao Paulo, Sao Paulo, SP, Brazil.

***Corresponding address:** Av. Dr. Arnaldo, 455, 3rd floor, room 3193, Sao Paulo, SP, 01246-903, Brazil | **Phone:** +55 11 3061-7490 | **Fax:** +55 11 3061-8595 | **E-mail:** [rosamariarp@yahoo.com](mailto:rosamariarp@yahoo.com)

**SUPPLEMENTARY TABLES:**

**S1**: Frequencies of isolated genotypes of *KLOTHO* SNPs C1818T, G395A, and C370S in the patient groups with osteopenia and osteoporosis at baseline.

|  | Osteopenia  (-1 >T-score >-2.5) | Osteoporosis  (T-score ≤ -2.5) |  |
| --- | --- | --- | --- |
| C1818T (rs564481) | n (%) | n (%) | P value |
| CC | 119 (41.5) | 130 (45.3) | 0.827 |
| CT | 102 (40.3) | 122 (48.2) |  |
| TT | 26 (43.3) | 29 (48.3) |  |
| Allele C | 221 (40.9) | 252 (46.7) | 0.652 |
| Allele T | 128 (40.9) | 151 (48.2) | 0.608 |
| G395A (rs1207568) |  |  |  |
| GG | 165 (40.5) | 192 (47.2) | 0.444 |
| GA | 80 (44.0) | 81 (44.5) |  |
| AA | 2 (18.2) | 8 (72.7) |  |
| Allele G | 245 (41.6) | 273 (46.3) | 0.210 |
| Allele A | 82 (42.5) | 89 (46.1) | 0.888 |
| C370S (rs9527025) |  |  |  |
| CC | 188 (40.3) | 219 (47.0) | 0.746 |
| CS | 59 (44.7) | 60 (45.5) |  |
| SS | 0 (0) | 1 (100.0) |  |
| Allele C | 245 (41.0) | 279 (46.9) | 0.620 |
| Allele S | 59 (44.0) | 61 (46.3) | 0.573 |

Data expressed as absolute frequency (n) and relative frequency.

Analyzed by Pearson χ^2^.

**S2**: Frequencies of isolated genotypes of *KLOTHO* SNPs C1818T, G395A, and C370S in the patient groups with clinical fractures, vertebral fractures and low appendicular muscle mass at baseline.

|  | **Clinical**  **fractures** |  | **Vertebral**  **fractures** |  | **Low**  **Appendicular Muscle Mass** |  |
| --- | --- | --- | --- | --- | --- | --- |
| **C1818T (rs564481)** | **n (%)** | **P-value** | **n (%)** | **P-value** | **n (%)** | **P-value** |
| **CC** | 33 (11.5) |  | 82 (29.4) |  | 48 (19.0) |  |
| **CT** | 25 (9.9) | 0.698 | 73 (29.8) | 0.948 | 38 (16.7) | 0.805 |
| **TT** | 8 (13.3) |  | 18 (30.5) |  | 10 (17.9) |  |
| **Allele C** | 58 (10.7) | 0.539 | 155 | 0.882 | 86 (17.9) | 0.997 |
| **Allele T** | 33 (10.5) | 0.720 | 91 | 0.886 | 48 (16.9) | 0.532 |
| **G395A (rs1207568)** |  |  |  |  |  |  |
| **GG** | 42 (10.3) |  | 120 (30.2) |  | 72 (19.8) |  |
| **GA** | 24 (13.1) | 0.302 | 50 (28.6) | 0.909 | 23 (14.0) | 0.242 |
| **AA** | 0 (0) |  | 3 (27.3) |  | 1 (11.1) |  |
| **Allele G** | 66 (11.2) | 0.240 | 170 (29.7) | .0.860 | 95 (18.0) | 0.593 |
| **Allele A** | 24 (12.4) | 0.452 | 53 (28.5) | 0.670 | 24 (13.9) | 0.095 |
| **C370S (rs9527025)** |  |  |  |  |  |  |
| **CC** | 57 (12.2) |  | 137 (30.3) |  | 78 (19.0) |  |
| **CS** | 9 (6.8) | 0.347 | 35 (27.1) | 0.351 | 18 (14.5) | 0.631 |
| **SS** | 0 (0) |  | 0 (0) |  | 0 (0) |  |
| **Allele C** | 66 (11.0) | 0.542 | 172 (29.7) | 0.889 | 96 (18.0) | 0.418 |
| **Allele S** | 9 (6.7) | 0.073 | 35 (27.5) | 0.533 | 18 (14.3) | 0.229 |

The values are expressed as the number of subjects and respective percentage (%).
